# A systematic review of school-based eHealth interventions targeting alcohol use, smoking, physical inactivity, diet, sedentary behaviour and sleep among adolescents: a review protocol

**DOI:** 10.1186/s13643-017-0645-x

**Published:** 2017-12-06

**Authors:** Katrina E. Champion, Nicola C. Newton, Bonnie Spring, Q. Eileen Wafford, Belinda J. Parmenter, Maree Teesson

**Affiliations:** 1 Department of Preventive Medicine, Northwestern University Feinberg School of Medicine, 680 N. Lake Shore Drive, Suite 1400, Chicago, IL 60611 USA; 2 NHMRC Centre of Research Excellence in Mental Health and Substance Use, NDARC, UNSW Sydney, Sydney, NSW Australia; 3 Galter Health Sciences Library, Northwestern University Feinberg School of Medicine, Chicago, IL USA; 4 School of Medical Sciences, UNSW Sydney, Sydney, NSW Australia

**Keywords:** Prevention, Risk, School, Adolescence, Alcohol, Smoking, Diet, Physical inactivity, Sedentary behaviour, Sleep

## Abstract

**Background:**

Six key behavioural risk factors (risky alcohol use, smoking, poor diet, physical inactivity, sedentary behaviour and unhealthy sleep patterns) have been identified as strong determinants of chronic disease, such as cardiovascular disease, diabetes and cancers. School-based interventions targeting these multiple health risk behaviours among adolescents have the potential to halt the trajectory towards later disease, whilst online and mobile technology interventions offer advantages in terms of student engagement, reach and scalability. Despite this, the efficacy of eHealth school-based interventions targeting these six health risk behaviours among adolescents has not been evaluated. The proposed systematic review aims to address this by determining the nature and efficacy of existing eHealth school-based interventions targeting multiple health risk behaviours among adolescents.

**Methods:**

A systematic search of the MEDLINE, Embase, PsycINFO and Cochrane Library databases will be conducted to identify eligible published papers. Eligible studies will be randomised controlled trials, including cluster randomised controlled trials, of interventions targeting two or more of the following lifestyle risk behaviours: alcohol use, smoking, poor diet, physical inactivity, sedentary behaviour and sleep. Eligible studies will be those evaluating interventions delivered in a secondary school setting among participants 11–18 years of age, via an eHealth platform (Internet, computers of mobile technology). Two reviewers will independently screen studies for eligibility, extract data and assess the risk of bias. Study outcomes will be summarised in a narrative synthesis, and meta-analyses will be conducted where it is appropriate to combine studies.

**Discussion:**

It is anticipated that the results from this review will serve to inform the development of future eHealth multiple health behaviour interventions for adolescents by identifying common characteristics of effective programs and highlighting knowledge gaps in the evidence base.

**Systematic review registration:**

PROSPERO CRD42017072163

**Electronic supplementary material:**

The online version of this article (10.1186/s13643-017-0645-x) contains supplementary material, which is available to authorized users.

## Background

Chronic diseases, such as cardiovascular diseases, diabetes and cancers, are the leading cause of death worldwide and are associated with significant costs and harms [1]. It is well established that the major chronic diseases share four common behavioural risks: poor diet, physical inactivity, smoking and alcohol use [2, 3]. In addition, there is now evidence that associates emerging risk behaviours, namely sedentary behaviour (i.e. sitting and screen time) [4, 5] and unhealthy sleep patterns (i.e. long or short duration, poor quality) [6], with chronic disease risk. Specifically, short sleep duration, poor quality and sleep timing (late bedtime or wake-up time) have been associated with poor health outcomes, such as obesity, among children and adolescents [7–10] and risk for later disease in adulthood [4, 6]. Similarly, studies investigating sedentary behaviour have demonstrated associations between screen time and markers of adiposity and cardiometabolic disease risk [11], mental health [12] and quality of life [13] in adolescents, and sedentary time has been linked to increased risk of all-cause, cardiovascular disease and cancer-related mortality, and incidence of these diseases, in adults [14, 15]. Sleep and sedentary behaviour are also important risk factors to consider given that they often co-occur, with other key risk behaviours in adolescence [10, 16, 17]. Recent research has also found a composite risk index encompassing these six behaviours (alcohol use, smoking, poor diet, physical inactivity, poor sleep and sedentary behaviour) to be highly predictive of all-cause mortality [4]. This association reinforces the importance of considering all six lifestyle risk factors in an effort to prevent future development of chronic disease.

 Risk behaviours commonly co-occur as clusters, as individuals engage in multiple risk behaviours concurrently [18]. This has prompted the development of multiple health behaviour change interventions [19], in which shared risk factors are targeted together, rather than in isolation. Many lifestyle risk behaviours emerge and develop in adolescence and then persist into adulthood; for example, dietary patterns established in adolescence continue into adulthood and are strongly associated with risk of heart disease later in life [20]. Conversely, research indicates that the adoption of a healthy lifestyle in adolescence can have protective effects against the onset of chronic disease [21]. Although chronic disease prevention should optimally occur at various stages across the life course, adolescence provides a critical opportunity to intervene *before* the onset of disease, thereby interrupting the long-term trajectory towards poor adult health. Multiple health behaviour change interventions have the potential to achieve this in an efficient and timely manner.

Secondary school is an ideal location for intervention delivery as educators can engage large numbers of students efficiently prior to risk behaviours becoming entrenched. Outside of the family environment, the school is the primary setting within which the development of children and young people can be directed and shaped [22] and common school and peer influences associated with lifestyle risk behaviours can be targeted [23]. The World Health Organization [24] recommends that schools include education about nutrition, physical activity and smoking to equip students with the knowledge and skills needed to prevent and manage chronic diseases, and the potential of the school environment for cardiovascular disease prevention is well supported [25]. Given that teaching time is often limited in school settings, interventions that can simultaneously address multiple risk behaviours are particularly advantageous. Additionally, eHealth interventions (i.e. those delivered via the Internet, computers or mobile technology) delivered in a school setting offer a number of potential advantages over traditional prevention programs, including increased student engagement, fidelity and scalability [26, 27]. Adolescents are extensive users of the Internet, with an estimated 97% of 15- to 17-year-olds [28] and 98% of 12- to 14-year-olds in Australia [29] accessing the Internet regularly. Internet technology is also becoming increasingly embedded in school education, with 86% of youth reporting using the Internet at school [30]. The use of mobile technology, such as smartphones, is also a commonplace among adolescents [31], and there is evidence to support the use of smartphone ‘apps’ to improve health behaviours in youth [32–34].

Previous systematic reviews of multiple health behaviour interventions have largely focused on adult populations [35–39], with less evidence among adolescents [23, 40–42]. Of the literature that does exist is a recent review by Hale and colleagues [23] which focused on interventions addressing tobacco, alcohol and illicit drug use; sexual risk behaviour; and aggressive behaviour among youth but did not assess the domains of diet, physical activity, sedentary behaviour or sleep. This review concluded that multiple health behaviour prevention programs are feasible and may be more efficient than prevention strategies targeting risk factors in isolation. The strongest effects were observed in relation to substance use outcomes and school-based settings. Another study [42] systematically reviewed interventions targeting sexual risk behaviour and substance use (alcohol, illicit drugs and tobacco) simultaneously among adolescents. This review found few studies and inconsistent effects, with multi-component school-based interventions seemingly offering the most promise. A third review of school-based multiple health behaviour interventions encompassed a broader range of risk factors including energy balance (diet, physical activity, screen time) and addiction (alcohol, drug and tobacco use) behaviours [43]; however, emerging risks, such as sleep and sitting time, were not included, and published literature beyond 2011 was not reviewed. Findings from this review suggest that too few studies of school-based multiple health behaviour interventions have been conducted to be able to determine whether targeting behaviours simultaneously has a synergistic effect.

Together, these reviews suggest that whilst there is a possibility that universal multiple risk behaviour interventions for young people are more efficient [23] and cost-effective [44], there is not yet strong evidence regarding whether they are effective and further research is needed. Furthermore, whilst previous reviews have examined eHealth interventions targeting various combinations of lifestyle risk behaviours among adult [35, 45] and youth [33, 34] populations, to our knowledge, there has been no systematic review of school-based eHealth interventions encompassing all six health risk behaviours in an adolescent population. To avoid duplication with a previously registered review protocol [46], and address gaps in the field, the proposed review aims to systematically review the literature on school-based eHealth interventions designed to target two or more of the following lifestyle risk behaviours among adolescents: alcohol use, smoking, poor diet, physical inactivity, sedentary behaviour and sleep. The specific objectives are to:

1. Determine the existence of school-based eHealth multiple health behaviour change interventions designed to target two or more of the six risk behaviours of interest
2. Evaluate the efficacy of existing school-based eHealth multiple health behaviour interventions in preventing alcohol use, smoking, poor diet, physical inactivity, sedentary behaviour and/or poor sleep among adolescents
3. Identify intervention characteristics (including duration, frequency, delivery mode, theoretical basis, type and number of risk behaviours targeted together) that are associated with effectiveness

A synthesis of the most recent evidence on eHealth multiple health behaviour interventions for adolescents will ideally guide the development of future interventions by identifying which combinations of risk behaviours have been effectively targeted simultaneously, as well as the content and delivery components associated with the effective interventions.

## Methods

This systematic review has been registered with the International Prospective Register of Systematic Reviews (PROSPERO; CRD42017072163) and was written in accordance with the Preferred Reporting Items for Systematic Review and Meta-Analysis Protocols (PRISMA-P) guidelines [47] as provided in Additional file 1. The planned systematic review will also be conducted in line with the PRISMA statement [48].

### Eligibility criteria

**Fig. 1:**
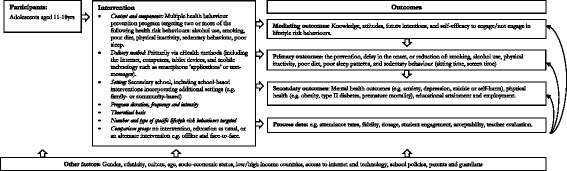
Logic model: eHealth school-based interventions targeting multiple health risk behaviours in adolescents

To be eligible for inclusion in the proposed systematic review, published studies must target adolescents aged between 11 and 18 years of age (i.e. those of secondary school age); evaluate a multiple health behaviour prevention program targeting two or more of the following health risk behaviours: alcohol use, smoking (including e-cigarette use), poor diet, physical inactivity, sedentary behaviour and poor sleep (duration and quality); and be primarily delivered via eHealth methods (including the Internet, computers, tablet devices and mobile technology such as smartphone ‘applications’ or text messages). Interventions must be conducted in a secondary school setting; however, school-based interventions incorporating additional components (such as family- or community-based elements) will also be eligible. Eligible study designs will be randomised controlled trials, including cluster randomised controlled trials. Studies with a comparison group that received no intervention, education as usual or an alternate intervention, including offline and face-to-face interventions, will be included. Programs must be universal in nature (i.e. delivered to all students regardless of their level of risk). Interventions addressing other risk behaviours in addition to two or more of the six behaviours of interest, for example, illicit drug use, risky sexual behaviour, sun protection habits and aggressive behaviour, will be eligible for inclusion. Whilst data for these additional outcomes will not be meta-analysed, studies and results may be discussed within the paper qualitatively. As recommended in the literature [49, 50], Fig. 1 displays the logic model for the proposed review.

### Search strategy

A librarian will develop a database search strategy in consultation with members of the review team. Databases to be searched will include Ovid MEDLINE, Embase (Elsevier), PsycINFO (EBSCOhost) and Cochrane Library (Wiley; an example search strategy for MEDLINE can be found in Additional file 2). The search will be limited to human research and to studies published between 2000 and 2017, given the focus on eHealth interventions; however, no language restrictions will be enforced. The search strategy will incorporate filters to identify randomised controlled trials. All papers identified in the search strategy will be exported into a citation management system (Endnote) for de-duplication and uploaded to the Covidence online software program for screening. The reference lists of eligible papers will be reviewed to identify other relevant studies, and recent related systematic reviews will be consulted to identify any additional studies. Grey literature, including clinical trial registries, will also be searched for unpublished studies, and conference proceedings/abstracts will also be reviewed.

### Data extraction and screening

The titles and abstracts of identified articles will be independently screened by two authors against the eligibility criteria, with any disagreement resolved by a third reviewer. Full-text copies of potentially relevant papers will be assessed for eligibility by the two reviewers. Data extraction will occur using a standardised extraction form, which will be piloted by the two reviewers to assure that it adequately captures trial data. Data will be extracted by two reviewers and will include:

- Publication details (study authors, year published)
- Study characteristics (design, country, sample size, attrition)
- Participant characteristics (e.g. age, gender, ethnicity, socio-economic status)
- Intervention characteristics (delivery method, program duration, frequency of delivery, theoretical basis, content and components, number and type of specific lifestyle risk behaviours targeted)
- Primary and secondary outcomes of interest across all time points
- Measurement tools employed (e.g. validated scales, objective measures)
- Details of the comparison group

Data to assess the risk of bias of each study and process data will also be extracted to determine the degree to which an intervention was implemented as intended (e.g. attendance rates, fidelity, dosage, student engagement). Where necessary, the corresponding author of included studies will be contacted by email to obtain any required data not presented in the published paper. Data will be entered in the review manager (RevMan) software for analysis.

### Outcomes

Primary outcomes of interest will be the prevention, delay in the onset, or reduction of any of the six lifestyle risk behaviours targeted in the intervention: smoking, alcohol use, physical inactivity, poor diet, poor sleep patterns and sedentary behaviour. Data for outcomes at all follow-up time points will be extracted and synthesised for all eligible studies. It is anticipated that there may be multiple measures of risk behaviours both across and within studies, for example lifetime alcohol use and binge drinking, and sleep duration and sleep quality. In these instances, all types and units of measurement of the lifestyle risk behaviour outcomes will be extracted. Secondary outcomes will include knowledge, attitudes, future intentions and self-efficacy to engage/not engage in the lifestyle risk behaviours, as well as mental (e.g. anxiety, depression, suicide or self-harm) and physical health outcomes (e.g. obesity, type II diabetes, premature mortality), educational attainment and employment.

### Risk of bias

Two reviewers will independently assess the risk of bias of the included studies using a modified version of the Cochrane Collaboration’s tool for assessing the risk of bias [51]. This tool covers a range of domains of potential bias, including sequence generation; allocation concealment; blinding of participants, personnel and outcome assessors; incomplete outcome data; selective outcome reporting; and any other threats to the validity of the trials (e.g. recruitment bias, baseline imbalance, incorrect analyses). Any discrepancies between the raters will be resolved by a third reviewer. Scores will be summed across the six domains to give a total score of the risk of bias for each study. Studies with a higher score will be deemed to be of higher quality; however, rather than focussing on just the scores, the quality of each study will be assessed by whether or not points were given for individual quality criterion. With randomisation already being a necessary criterion, studies that have points allocated for allocation concealment, blinding of participants and outcome assessors, and intention to treat analysis and are free of any other bias will be deemed to be of higher quality and therefore lower risk of bias.

### Analysis

We will conduct a narrative synthesis on all available data. A qualitative synthesis of the following study aspects will be conducted: intervention content (i.e. risk behaviours targeted), delivery method, intervention frequency and duration, and sample characteristics (e.g. age). A quantitative analysis of all primary outcomes where enough data is available will also be conducted. We anticipate that there is likely to be a high degree of heterogeneity with respect to participant age, intervention types/lengths, reporting of outcomes and outcome measurements. Therefore, if it is appropriate to combine studies, we will conduct an inverse variance random-effects analysis on each of the outcomes. The random-effects analysis may then account for any differences in outcome measure results and sample size across the studies. Inconsistency between groups will be quantified using Higgins *I* ^2^, with scores ranging from 0 to 100%. Higgins *I* ^2^ represents the total variation that is attributed to the true difference between the studies, with values > 50% possibly representing substantial heterogeneity. The significance of any heterogeneity identified will be examined using the Cochran’s *Q* (chi^2^) test with *p* < 0.05 indicating significant heterogeneity. If significant heterogeneity is present, then a sub-analysis on outcomes by elements of the interventions (e.g. type of eHealth intervention: online, smartphone, other; duration/setting of intervention; number and type of behaviours targeted; gender; age of participants; and low/high-income countries) may be warranted to identify sources of heterogeneity. Sensitivity analyses may be used to restrict analyses to studies at low risk of bias, lower age groups, socioeconomic status and/or where other issues suitable for sensitivity analysis have been identified during the review process. In addition, funnel plots will be visually examined for publication bias. We will use the Grading of Recommendations Assessment, Development and Evaluation (GRADE) framework to assess the quality of the body of evidence [52].

## Discussion

The proposed systematic review will be the first to evaluate the efficacy of eHealth school-based multiple health behaviour interventions designed to prevent, delay the onset or reduce six key lifestyle risk behaviours among adolescents: alcohol use, smoking, diet, physical inactivity, sedentary behaviour and poor sleep. A systematic review of the most recent evidence will serve to inform the development of future eHealth interventions addressing multiple risk behaviours among secondary school students. Interventions designed to address these lifestyle risk factors among adolescents, which are rendered scalable and engaging through their Internet- or mobile-based delivery, have the potential to produce short-term improvement in young peoples’ health and also to reduce the accumulation of risk for later chronic disease in adulthood.

## Additional files

Additional file 1: PRISMA-P checklist. This document entails a completed PRISMA-P checklist. (DOCX 29 kb)

Additional file 2: Search strategy. This document provides an example search strategy. (DOCX 15 kb)

## Abbreviations

GRADE: Grading of Recommendations Assessment, Development and Evaluation
PRISMA-P: Preferred Reporting Items for Systematic Review and Meta-Analysis Protocols
PROSPERO: International Prospective Register of Systematic Reviews
