# Supplementary material for: A systematic review of school-based eHealth interventions targeting alcohol use, smoking, physical inactivity, diet, sedentary behaviour and sleep among adolescents: a review protocol
Source: Syst Rev. 2017 Dec 6;6:246. doi: 10.1186/s13643-017-0645-x (PMC5717802; doi:10.1186/s13643-017-0645-x)
Supplement: Supplementary file 2 — Search strategy. This document provides an example search strategy. (DOCX 15 kb) [file 13643_2017_645_MOESM2_ESM.docx]

Search Strategy

Database(s): **Ovid MEDLINE(R) In-Process & Other Non-Indexed Citations and Ovid MEDLINE(R)**1946 to Present 
Search Strategy: September 1, 2017

| **#** | **Searches** | **Results** |
| --- | --- | --- |
| 1 | exp Adolescent/ | 1884600 |
| 2 | exp Child/ | 1790983 |
| 3 | (adolescen* or girl* or boy* or youth* or child or children or teen* or minors or juvenil* or pubert* or pubescen* or pre-pube* or kid or kids or underage*).tw. | 1421750 |
| 4 | 1 or 2 or 3 | 3224583 |
| 5 | Students/ | 48028 |
| 6 | Schools/ | 32257 |
| 7 | exp Education/ | 682232 |
| 8 | (school* or teacher* or classroom* or educat* or student* or curricul*).tw. | 818918 |
| 9 | 5 or 6 or 7 or 8 | 1199703 |
| 10 | exp Drinking Behavior/ | 68782 |
| 11 | (alcoholic* or intoxication or liquor or hangover*).tw. | 96581 |
| 12 | (alcohol* adj3 (abus* or us* or disorder* or consum* or drink* or heav* or problem* or excess* or bing* or risk* or reckless*)).tw. | 111499 |
| 13 | (drink* adj3 (alcohol* or abus* or us* or disorder* or consum* or heav* or problem* or excess* or bing* or risk* or reckless*)).tw. | 37293 |
| 14 | 10 or 11 or 12 or 13 | 229536 |
| 15 | exp Smoking/ | 141619 |
| 16 | exp "Tobacco Use Disorder"/ | 10458 |
| 17 | exp "Tobacco Use Cessation"/ | 26549 |
| 18 | (cigarette* or smok* or tobacco or vaping or vape* or eCig* or e-cig*).tw. | 290955 |
| 19 | 15 or 16 or 17 or 18 | 323780 |
| 20 | exp Sports/ | 165014 |
| 21 | Motor Activity/ | 94873 |
| 22 | exp Exercise/ | 163117 |
| 23 | exp Physical Exertion/ | 58910 |
| 24 | exp "Play and Playthings"/ | 12198 |
| 25 | exp Dancing/ | 2545 |
| 26 | Recreation/ | 6389 |
| 27 | (fitness or exercis* or recess or sport* or game* or aerobic* or running or walking or jumping or outdoor* or "energy balance").tw. | 563303 |
| 28 | (physical* adj2 (activ* or inactivit* or energy or education)).tw. | 97582 |
| 29 | (insufficient* adj2 (activ* or energy or education)).tw. | 2084 |
| 30 | 20 or 21 or 22 or 23 or 24 or 25 or 26 or 27 or 28 or 29 | 807483 |
| 31 | exp Nutritional Physiological Phenomena/ | 542598 |
| 32 | exp "Diet, Food, and Nutrition"/ | 1564354 |
| 33 | exp Diet/ | 249469 |
| 34 | exp Feeding Behavior/ | 153824 |
| 35 | exp Nutrition Disorders/ | 318988 |
| 36 | (diet* or eat* or nutrition* or meal* or food* or lunch* or breakfast* or dinner* or supper or snack* or fruit* or vegetable* or sugar* or salt* or appetite* or overeat* or over-eat* or overnutrit* or over-nutrit* or overweight or obese or obesity or overnourish* or over-nourish*).tw. | 1475541 |
| 37 | 31 or 32 or 33 or 34 or 35 or 36 | 2574501 |
| 38 | exp Sedentary Lifestyle/ | 6311 |
| 39 | exp Television/ | 31250 |
| 40 | exp Leisure Activities/ | 212493 |
| 41 | (sedentary or sitting or television or (video adj game*) or (web adj2 brows*) or (watch* adj2 TV)).tw. | 57897 |
| 42 | ((electronic* or computer* or media) adj2 (game* or us* or play* or time*)).tw. | 60915 |
| 43 | (screen adj2 (time* or recreation* or view* or activit*)).tw. | 2022 |
| 44 | 38 or 39 or 40 or 41 or 42 or 43 | 344383 |
| 45 | exp Sleep/ | 72138 |
| 46 | (sleep* or insomnia*).tw. | 157444 |
| 47 | 45 or 46 | 174760 |
| 48 | exp Telecommunications/ | 81208 |
| 49 | exp Computing Methodologies/ | 852264 |
| 50 | Medical Informatics Applications/ | 2431 |
| 51 | Decision Making, Computer-Assisted/ | 2757 |
| 52 | exp "Information Storage and Retrieval"/ | 147730 |
| 53 | exp Information Systems/ | 213398 |
| 54 | exp Communication/ | 443456 |
| 55 | exp Technology/ | 373438 |
| 56 | (eHealth or android* or app or apps or audio* or blog* or CD-ROM* or cellphone* or (cell* adj phone*) or computer* or cyber* or DVD* or "e-health" or (electronic* adj health) or e-Portal* or ePortal* or (electronic* adj portal*) or eTherap* or e-therap* or (electronic* adj therap*) or forum* or gaming or (information adj technolog*) or (instant adj messag*) or internet* or ipad* or i-pad* or iphone* or i-phone* or ipod* or i-pod* or laptop* or (live adj chat*) or web* or WWW or (smart adj phone*) or smartphone* or ("social network" adj1 site*) or (mobile adj phone*) or e-mail* or email* or mHealth or m-health or mobile* or multi-media or multimedia or online* or on-line or SMS or ("short message" adj service*) or software or telecomm* or telehealth* or telemed* or telemonitor* or telephone or teletherap* or (text adj messag*) or texting or podcast* or virtual* or media or digital* or technolog* or remote* or computer* or web or online or computeri?ed or pedomet* or (electronic adj application*) or Kindle* or ("personal digital" adj assistant*)).tw. | 1533523 |
| 57 | (Facebook or WhatsApp or YouTube or skype or Instagram or twitter or Tumblr or Snapchat or Reddit or Myspace).tw. | 4167 |
| 58 | 48 or 49 or 50 or 51 or 52 or 53 or 54 or 55 or 56 or 57 | 2821182 |
| 59 | 4 and 9 and 58 and (14 or 19 or 30 or 37 or 44 or 47) | 20306 |
| 60 | ((randomized controlled trial or controlled clinical trial).pt. or randomized.ab. or randomised.ab. or placebo.ab. or clinical trials as topic.sh. or random*.ab. or trial.ti.) not (exp animals/ not humans.sh.) | 1249565 |
| 61 | 59 and 60 | 4172 |
| 62 | limit 61 to yr="2000 - 2017" | 3706 |
